# Rapid emergence of *Trichophyton indotineae* (*Trichophyton mentagrophytes* ITS genotype VIII) observed in the United Kingdom, up to August 2025

**DOI:** 10.2807/1560-7917.ES.2025.30.49.2500892

**Published:** 2025-12-11

**Authors:** Alexandra Czerniewska, Andrew Borman, Alireza Abdolrasouli, Emma L Budd, Matthew C Fisher, Richard Barton, Kieran A Bates, Jonathan Lambourne, Alicia Demirjian, Berit Muller-Pebody, Colin S Brown, Maria Saavedra-Campos, Rohini Manuel

**Affiliations:** 1 Field Epidemiology Training Programme, UK Health Security Agency, London, United Kingdom; 2 National Mycology Reference Laboratory, UK Health Security Agency, Bristol, United Kingdom; 3 MRC Centre for Medical Mycology, University of Exeter, Exeter, United Kingdom; 4 AMR & HCAI Division, UK Health Security Agency, London, United Kingdom; 5 Department of Infectious Disease Epidemiology, Imperial College London, London, United Kingdom; 6 Mycology Reference Centre, Leeds Teaching Hospitals NHS Trust, Leeds, United Kingdom; 7 Blizard Institute, Queen Mary University of London, London, United Kingdom; 8 Department of Infection, Barts Health NHS Trust, London, United Kingdom; 9 Department of Paediatric Infectious Diseases & Immunology, Evelina London Children's Hospital, London, United Kingdom; 10 Faculty of Life Sciences & Medicine, King’s College London, London, United Kingdom; 11 Field Services Southeast and London, UK Health Security Agency, London, United Kingdom

**Keywords:** Trichophyton, Indotineae, Tinea, Dermatophyte, Arthrodermataceae, Mycology

## Abstract

*Trichophyton indotineae* is an emerging dermatophyte increasingly detected in the United Kingdom, often with antifungal resistance. We identified 363 cases between January 2017 and August 2025, with 310 cases (85%) since January 2023. Cases were mostly concentrated in major urban centres and most affected individuals were working-age adults, frequently reporting South Asian ethnicity. The importance of international importation vs domestic transmission remains unclear. Implementing enhanced surveillance would help to identify risk factors and transmission routes to focus prevention efforts.

In May 2025, the United Kingdom (UK) Health Security Agency (UKHSA) cascaded an official notification to inform all clinicians and laboratory staff of the emergence of cases of *Trichophyton indotineae. Trichophyton indotineae* (previously named *Trichophyton mentagrophytes* ITS genotype VIII) is an emerging anthropophilic dermatophyte associated with persistent skin infections (tinea), high relapse rates and social stigma [1-3], and resistance to antifungals. Here, we report the rapid and continuing increase in new *T. indotineae* detections in the UK up to August 2025, extending a previous analysis [4], and highlighting key challenges and opportunities for surveillance and research.

## Incidence of confirmed *Trichophyton indotineae* infections 2017–2025

All UK laboratories, including regional mycology laboratories, are encouraged to submit confirmed or suspected *T. indotineae* isolates to the central UKHSA mycology reference laboratory (MRL) for confirmation and epidemiological tracking on a voluntary basis (without cost to the referring laboratory for confirmatory identification). We retrospectively reviewed all *T. indotineae* isolates confirmed by the MRL between 1 January 2017 and 22 August 2025, using previously described methods [4]. We deduplicated MRL data, keeping the first positive sample per individual (new cases).

**Figure 1:**
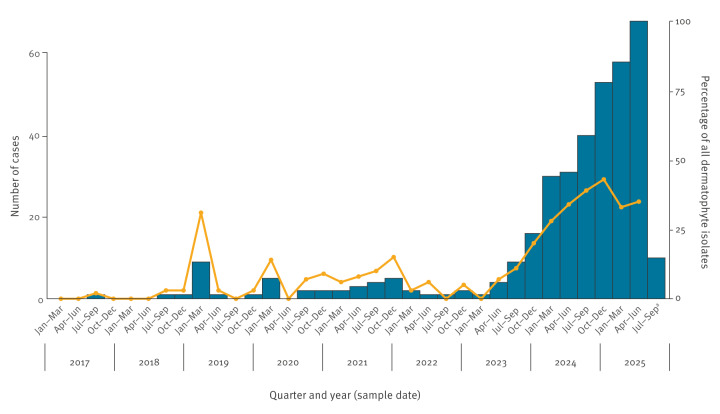
Quarterly distribution of *Trichophyton indotineae* cases referred to the UKHSA mycology reference laboratory, United Kingdom, January 2017–August 2025 (n = 363)

There were 363 new cases recorded over this period. Between 2017 and 2022, incidence was low and stable, with one to 15 cases detected annually; quarterly incidence rose rapidly from one case in January–March 2023 to 69 in April–June 2025 (Figure 1). *Trichophyton indotineae* isolates accounted for > 30% of all dermatophyte isolates referred to the MRL in each quarter since the second quarter of 2024.

UKHSA: United Kingdom Health Security Agency.

^a^ Data collection for the quarter July–September 2025 was incomplete at the time of analysis.

*Trichophyton indotineae* cases (n = 363) are presented by first sample date or date received by the UKHSA mycology reference laboratory (MRL), if sample date was unavailable (blue bars). The proportion of dermatophyte isolates processed at the MRL that were identified as *T. indotineae,* by date received at MRL, is indicated by the yellow line.

## Characteristics of *Trichophyton indotineae* cases

**Figure 2:**
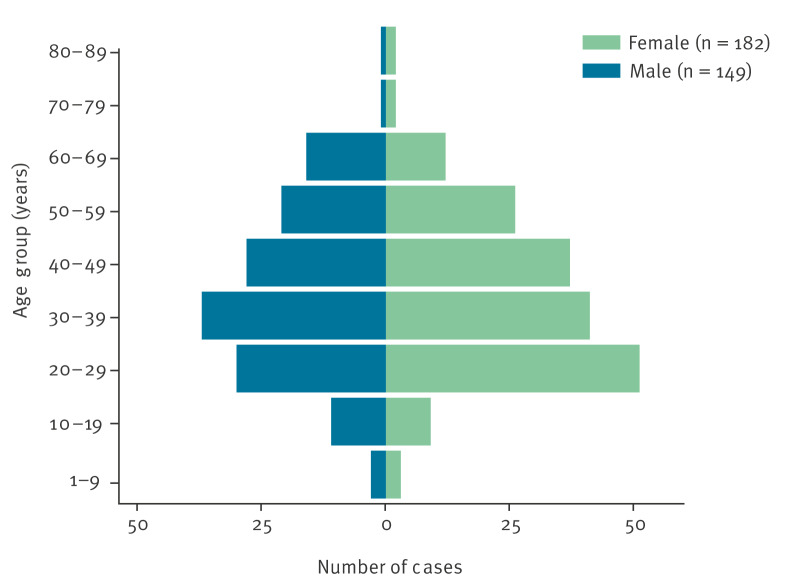
Age and sex distribution of *Trichophyton indotineae* cases referred to the UKHSA mycology reference laboratory, United Kingdom, January 2017–August 2025 (n = 331)

We extracted structured and unstructured demographic, clinical and epidemiological data provided by clinicians through the laboratory requisition forms submitted with isolates. The median age of affected individuals was 36 years (range: 2–82 years), with 80% of cases occurring in adults aged 20–59 years (n = 292). Among 331 cases (91%) with recorded sex, 182 (55%) were female and 149 (45%) were male (Figure 2). According to the clinical notes provided with samples submitted to the MRL, some individuals had a history of tinea symptoms lasting several years before a sample was obtained.

UKHSA: United Kingdom Health Security Agency.

Of the 363 total cases, 32 cases with unknown sex were excluded.

Of 220 cases (61%) where a specific affected body site was documented, the majority (62%, n = 137) had tinea cruris infection in the groin, buttocks or thigh area (Table). As previously described [4], a subset of clinical isolates collected up to May 2024 (n = 124) were subjected to antifungal susceptibility testing using broth microdilution method, showing 74% (92/124) had raised MIC of > 0.5 mg/L to terbinafine.

**Table:** Characteristics of *Trichophyton indotineae* cases referred to the UKHSA mycology reference laboratory, United Kingdom, January 2017–August 2025 (n = 363)

Characteristics	*T. indotineae* cases (n)	%
Anatomical site affected^a^		
Buttock, groin, gluteal fold, perineum, thigh	137	38
Back, abdomen, torso, trunk, breast, chest	49	14
Legs, feet, knee, toenail	31	9
Arms, hands, axilla	28	8
Face, neck, head	10	3
Unknown	143	39
Self-reported ethnicity^b^		
Bangladeshi (Asian or Asian British)	74	40
Indian (Asian or Asian British)	38	21
Pakistani (Asian or Asian British)	20	11
Any other Asian background	19	10
British (White)	18	10
Any other ethnic group	7	4
African (Black or Black British)	3	2
Any other White background	2	1
Any other Mixed background	1	0
White and Asian (mixed)	1	0
Unknown	180	50

UKHSA: United Kingdom Health Security Agency.

^a^ Multiple sites reported in 33 cases.

^b^ Ethnicity categories reported through the United Kingdom national health system (NHS).

We were able to confirm the self-reported ethnicity for 183 cases (50%) provided during any hospital admission between 2000 and 2025, through linkage to the NHS Hospital Episodes Statistics (HES) database [5]. Among these, 83% (n = 151) reported an Asian or Asian British ethnicity (Table). Travel history — either by the case, a partner or through an unspecified epidemiological link — was reported through the MRL for 41 cases (11%), with 39 of these associated with a South Asian country (15 of the 41 cases also reported an Asian or Asian British ethnicity; 5 reported another ethnicity and 21 had no ethnicity data available).

There were 10 case clusters sharing the same postcode (nine pairs and one trio; 21 cases total). Seven clusters involved individuals from the same household, while the remaining three were from closely located households. Clinical notes also indicated five additional cases with affected household members not included in the dataset, as no isolates were submitted for them.

## Geographical distribution

**Figure 3:**
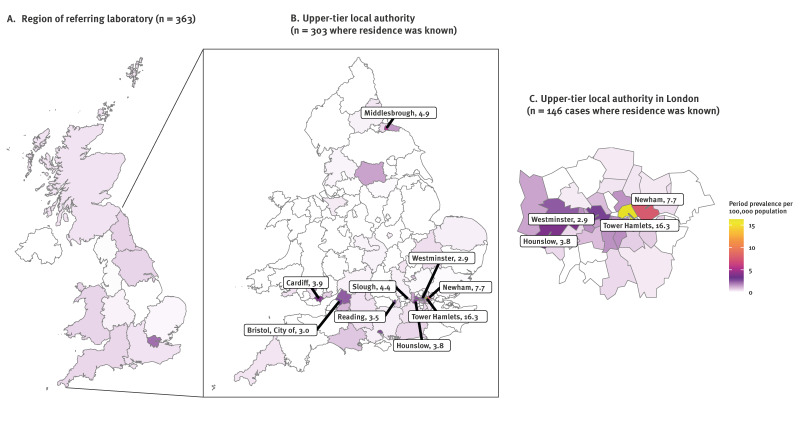
Period prevalence rate of *Trichophyton indotineae* cases per 100,000 population referred to the UKHSA mycology reference laboratory, United Kingdom, January 2017–August 2025

Isolates were referred to the MLR from laboratories across 10 of the 12 UK regions. We mapped cases resident in England and Wales to their local government area (upper tier local authority) of residence (Figure 3). Cases were distributed across 66 of 144 (46%) local areas. Notably, 84% of mapped cases (256/303) resided in areas classified as ‘major towns and cities’ [6] compared with ca 50% of the general population.

Panel A. Regions of referring laboratories across the United Kingdom, shaded to indicate referral activity levels. Panel B. Upper-tier local authorities of residence in England and Wales for cases identified in 2025. Labels are provided for areas with a period prevalence ≥ 3 per 100,000 population. Panel C. London region, highlighting boroughs with highest prevalence rates. Source: UKHSA Mycology Reference Laboratory.

We calculated the period prevalence for each area using official 2021 population statistics [7]. The three highest prevalence rates were observed in two areas of London, i.e. Tower Hamlets and Newham with 16.3 and 7.7 cases per 100,000 population, respectively, and one area in North-East England, Middlesbrough with 4.9 cases per 100,000 population.

## Discussion

In the past 2 decades, severe or antifungal-resistant dermatophytoses have become a global public health concern [8]. *Trichophyton indotineae* is of particular importance due to its documented resistance to terbinafine, a first-line antifungal treatment for dermatophyte infections, which complicates and prolongs infections and imposes a substantial burden on patients’ quality of life. First identified in South Asian and Middle Eastern countries in the early 2010s [9,10], genomic analyses have confirmed its rapid, transcontinental clonal dissemination, and this dermatophytosis is now reported worldwide [11,12]. 

The observed increase in *T. indotineae* cases in the UK between 2017 and 2025 mirrors that seen in several other countries, including Germany which recorded 233 cases and a similarly sharp rise across a comparable period [13,14]. The increase in UK cases likely reflects a combination of a true rise in incidence coupled with greater awareness among patients, medical practitioners and microbiology laboratorians to recognise cases and refer isolates.

*Trichophyton indotineae* is believed to predominantly spread from person-to-person [9], but there is little published evidence on the age and sex of those most affected or the main risk factors for acquisition. Our findings concur with data from Germany and Canada showing that cases occurred mostly in working-age adults [14,15], although we observed a greater proportion of female cases (55% female in UK vs 31% female in Germany). The frequent involvement of the groin area in infections leads us to hypothesise common transmission pathways through bed sharing, sexual contact, sharing of towels or contact with shared surfaces. It is possible that these pathways are of differing importance in different settings. For example, some countries have reported clusters of *T. indotineae* and the closely related *T. mentagrophytes* genotype VII associated with sexual transmission [16], including among sex workers and men who have sex with men [17-19]. Autochthonous sexual transmission has not yet been documented in the UK for *T. indotineae*, which could in part explain why the sex distribution differs from that reported in other countries. However, the recognised limitations of the dataset make it difficult to draw conclusions with certainty.

Understanding the balance between imported and locally acquired cases is key to guiding an effective public health response. Evidence of widespread local transmission would highlight the need for strengthened clinical guidance and targeted interventions to prevent *T. indotineae* from becoming the dominant cause of dermatophytosis, as seen in India and Iran [20,21]. In our UK dataset, as with data from Germany [13], most cases identified as British Asian, South Asian, and South-east Asian, potentially indicating epidemiological links with endemic countries. While some clustering of cases within households was observed, data were not systematically collected to distinguish intra-household transmission from households travelling together. Enhanced surveillance through simple questionnaires distributed in primary care, or retrospectively sent following MRL confirmation, could help establish whether the UK has sustained community transmission.

We observed variation in prevalence nationwide, with notably low rates in some cities with a population density and socioeconomic and ethnic profiles similar to higher-prevalence areas. Future studies will determine whether this reflects differences in clinical recognition and referral practices, or true differences in prevalence.

This analysis had several limitations. Firstly, our data likely underestimate the true number of cases because of delayed diagnosis and under-ascertainment; skin scrapings are not always taken from cases upon examination and even if taken, may have a low yield. Secondly, our analysis relied on voluntary isolate referrals, but isolates may have been missed for a variety of reasons such as staff not recognising them in the laboratory. Finally, laboratory requisition forms often lack complete information, especially related to travel. While linking laboratory, clinical and epidemiological data offered valuable insights into case location and ethnicity, incomplete linkage limited our ability to classify and analyse all cases accurately.

## Conclusion

We observed rapid emergence of *T. indotineae* infections in the UK since the beginning of 2023. Where data were available, we found that most cases were in populations with plausible links to endemic countries, although travel histories were not usually confirmed. Given global experience, sustained local transmission seems highly likely, highlighting the urgent need for comprehensive surveillance and epidemiological research to clarify prevalence, transmission dynamics and risk factors. Achieving this will require increased clinical and laboratory awareness, improved referral pathways and enhanced case data.

## Data Availability

The data that support the findings of this study are available upon request, subject to approval, to the corresponding author.
